# Caffeine Effects on Physical Performance and Sport-Specific Skills in Elite Youth Soccer Players: A Randomised Trial Using the Balanced Placebo Design

**DOI:** 10.3390/sports13040106

**Published:** 2025-03-31

**Authors:** Eduard Bezuglov, Timur Vakhidov, Ryland Morgans, Georgiy Malyakin, Anton Emanov, Egana Koroleva, Elizaveta Kapralova, Oleg Talibov

**Affiliations:** 1 Department of Sports Medicine and Medical Rehabilitation, Sechenov First Moscow State Medical University, 119991 Moscow, Russia; bezuglov_e_n@staff.sechenov.ru (E.B.); vakhidovsc@yandex.ru (T.V.); rmorgans@cardiffmet.ac.uk (R.M.); kapralova_e_s@staff.sechenov.ru (E.K.); 2 High Performance Sports Laboratory, Sechenov First Moscow State Medical University, 119991 Moscow, Russia; 5184436@gmail.com (A.E.);; 3 Therapy, Clinical Pharmacology and Ambulance Care Department, Russian University of Medicine, 127006 Moscow, Russia; oleg.talibov@gmail.com

**Keywords:** young athletes, placebo effect, repeated sprint ability, football, sprint, strength

## Abstract

The aim of the study was to examine the safety and effects of relatively high caffeine doses on physical performance and sport-specific skills of young elite soccer players. Fifty-four soccer players from an elite Russian soccer academy participated in the blinded randomised placebo-controlled study with a double-blinded and balanced design. Participants were divided into four groups: group 1 CAF/CAF; group 2 CAF/PLA group 3 PLA/PLA and group 4 PLA/CAF. All participants were administered 400 mg of caffeine or the placebo. The data demonstrated that a single 400 mg caffeine administration 60 min before exercise had a positive effect on repeated sprint ability (RSA) test parameters such as fatigue index (for both groups *p* < 0.001) and percentage decrement score (for both groups *p* < 0.001). In group 4, statistically significant improvements were also found when performing the fourth and sixth sprint during the RSA test (*p* = 0.039 and *p* = 0.005, respectively). Group 1 also showed a statistically significant improvement in dribbling speed performance (*p* = 0.048). It was demonstrated that the number of adverse events in all four groups was not different (*p* = 0.56). A single administration of 400 mg of caffeine 60 min prior to maximal intensity physical activity can be considered reasonable and safe for young elite soccer players.

## 1. Introduction

Modern professional soccer places great demands on the human body, primarily due to the constantly increasing level of competition and the requirement to maintain a high level of physical and sport-specific qualities [1]. Moreover, young players of leading academies begin to fully experience competition as early as 11–12 years of age participating on a weekly basis in match-play on soccer fields.

In this regard, the issue of improving and maintaining the key physical qualities for soccer at different stages of the career plays a major role. Undoubtedly, the basis of sporting success is talent aligned with an appropriate athlete development programme, which includes adequate training volume and intensity and age-specific optimisation as well as the creation of a favorable socio-cultural environment around the athlete [2]. Another significant factor contributing to the success of soccer players is the use of various aids and methods to improve physical performance and optimise post-load recovery [3]. As the game is becoming increasingly faster at the elite level regardless of age and more intense due to a large number (60 and more) of competitive matches during the season, an interest in various aids to support these demands is also increasing. Due to the growing risk of over-training and injury and subsequent financial burden to clubs, the main aim of these various ergogenic aids is to improve physical soccer performance through single-dose or course use in different phases of training and competitive processes [4,5,6,7].

However, many of these substances (e.g., anabolic agents, metabolic modulators, peptide hormones) are banned by the World Anti-Doping Agency’s Prohibited List, which includes substances and/or methods with potentially positive effects on sport performance and/or potentially negative effects on athlete health. However, the list of non-prohibited dietary supplements with proven positive effects on physical performance is very limited and currently includes only a few substances such as beta-alanine, nitric oxide precursors (beetroot juice, citrulline, arginine), sodium bicarbonate, creatine and caffeine [8,9]. One of the most popular and well-studied substances is caffeine which is often consumed by large cohorts of athletes in various forms. There are numerous studies examining both the efficacy and safety of various doses of caffeine across a wide range of samples [10,11,12,13]. It should be noted that the ergogenic effect of caffeine may be due to its direct mechanism of action as well as the expectation of its effects, especially in individuals who have previously had a positive experience with its use. However, there are still no studies conducted in elite young soccer players that have evaluated the effect and safety profile of various doses of caffeine in the context of soccer-specific maximal intensity exercise, as well as the associated placebo effect. Thus, this topic is of great practical interest and will allow the effect of caffeine to be quantified to improve sport-specific performance and success of soccer players, and to develop an effective and safe protocol for its use. Therefore, the aims of this study were to examine the safety and effects of relatively high caffeine doses (400 mg) on physical performance and sport-specific skills of young elite soccer players. The study hypothesis was that caffeine would have a greater effect on soccer-specific skills compared to physical tests.

## 2. Materials and Methods

### 2.1. Participants

This blinded randomised placebo-controlled study with a double-blinded and balanced design involved 60 young elite soccer players from three teams (U15–U17) of the country’s leading soccer academy. Only 54 participants ((median; IQR; min–max) age 16.5; 1.2; 15.1–17.8 years, height 180; 9.5; 157–199 cm, body mass 69.2; 9.97; 46.4–94.8 kg, BMI 21.4; 2.07; 17.9–23.9 kg/m^2^, somatic maturation 98.6; 2; 91–100) completed the study. From the sample cohort, 48 were outfield players and six were goalkeepers.

The study inclusion criteria were participation in regular soccer training for at least six years, consistent member of elite youth academy soccer and experience in the tests utilised in the study. The exclusion criteria included the following:

- The presence of injuries and illnesses that caused the missing of more than three training sessions within three months prior to the time of the study;
- Refusal to participate in the study at any stage;
- An anxiety score above 10 points on the GAD-7 questionnaire;
- An injury sustained during the study that prevented full study completion;
- Any allergic reactions linked to caffeine in the participant’s medical history or during the study;
- Administration of medications potentially affecting the pharmacokinetics or pharmacodynamics of caffeine within 24 h prior to the start of the study;
- Administration of any other ergogenic substance in the 48 h prior to the start of the study;
- Smoking and use of psychoactive substances within 72 h before the start of the study.

### 2.2. Experimental Design

All participants were tested two separate times on control and experimental days at the same time, which were seven days apart. On the experimental day, each participant was given either caffeine presented as caffeine or placebo or placebo presented as placebo or caffeine. Participants were familiar with all the tests and had performed them at least twice in the past six months.

All tests were performed between 11:00 a.m. and 3:00 p.m. indoors (temperature 21–23 degrees Celsius, humidity 45–50%) on an artificial soccer surface habitual to athletes. Participants were dressed in their regular training kit (shorts, t-shirt and boots).

In the 48 h prior to testing, participants had no intense exercise (there were either two days of rest or one day of rest plus one day of light exercise) and were asked to abstain from using caffeine-containing products and any ergogenic aids for 48 h prior to testing. All participants were advised to adhere to their normal diet, which included a standardised breakfast at least 3 h prior to testing. None of the participants consumed nicotine, psychoactive drugs, or other drugs that could affect the pharmacokinetics and pharmacodynamics of caffeine within 48 h before the study.

On the control day, the Generalised Anxiety Disorder Questionnaire (GAD-7) and the Caffeine Consumption Questionnaire-Revised (CCQ-r) were completed before the physical performance and sport-specific skills tests. All participants were instructed on the rules for completing the questionnaires. A study coordinator was present with the participants to ensure that the questionnaires were completed correctly.

On the experimental day before taking caffeine or placebo capsules, all participants were informed by the coordinator (either researcher or sports medicine physician) that caffeine is a safe substance commonly used by elite athletes for its proven positive effects on athletic performance.

### 2.3. Participant Groups

Using block randomisation with stratification by age and field position (outfield player or goalkeeper), participants were divided into four groups:

- Group 1 (n = 14)—CAF/CAF: received caffeine and were informed that they had received caffeine;
- Group 2 (n = 12)—CAF/PLA: received placebo and were informed that they had received caffeine;
- Group 3 (n = 15)—PLA/PLA: received placebo and were informed that they had received placebo;
- Group 4 (n = 13)—PLA/CAF: received caffeine and were informed that they had received placebo.

Although the randomisation plan involved assigning 60 participants to four groups (15 in each), six participants dropped out during the screening phase. Randomisation was performed within strata, so the final number of participants in the groups appeared to vary. No participant replacements were planned due to the specific and limited population (highly trained soccer players who are members of the same club).

### 2.4. Patient and Public Involvement Statement

Athletes were not involved in research design or the outcome measures, but were intimately involved in setting the research question as the players were curious about safety and effectiveness of caffeine. Participants were important to dissemination of the baseline information, which helped to motivate community involvement during and beyond the study.

### 2.5. Equity, Diversity and Inclusion Statement

The research team included 6 men and 2 women from different disciplines (clinical medicine, pharmacology, sports medicine and epidemiology), including four authors considered junior scholars. The study population included youth male soccer players with different socioeconomic status. However, we acknowledge that we did not examine the effects of sex, race and ethnicity.

### 2.6. Standing Height and Body Mass

Standing height was measured to the nearest 0.1 cm using a portable stadiometer (Seca, model 217, Gamburg, Germany). Participants’ height was assessed in strict accordance with the rules of the The International Society for the Advancement of Kinanthropometry by a trained specialist. The participants stood with their feet together and the heels, buttocks and upper part of the back touching the stadiometer. The measurer placed the hands along participants’ jaw with the fingers reaching to the mastoid processes. The participant was instructed to take a deep breath and hold, and while keeping the head in the Frankfort plane, the measurer applied a gentle upward lift through the mastoid processes. Another member of staff placed the head-board firmly down, compressing the hair as much as possible. Measurement was taken at the end of a deep inspiration hold [14].

Body mass can be affected by hydration status and prolonged standing [15]. In an effort to minimise these effects, body mass measurements for each participant were obtained on a single day between 08:00–12:00. Participants were asked not to eat after 22:00 the night before testing and to refrain from exercise for 12 h prior to testing. Participants came to the laboratory refraining from exercise and/or stimulant beverages and fasting for at least 3 h. Body mass was measured using a calibrated electronic scale without shoes and wearing minimal clothes, to the nearest 0.01 kg according to standardised procedures described elsewhere [16]. Floor scales Seca-813 (Seca, Germany) were used for body mass measurement. Body mass measurements were performed in shorts and T-shirt only without shoes. The height of the biological parents for its subsequent use in the Hamis Roche formula was determined prior to the study by telephone or personal contact.

### 2.7. Physical Performance and Sport-Specific Skills Test Protocols

**Figure 1:**
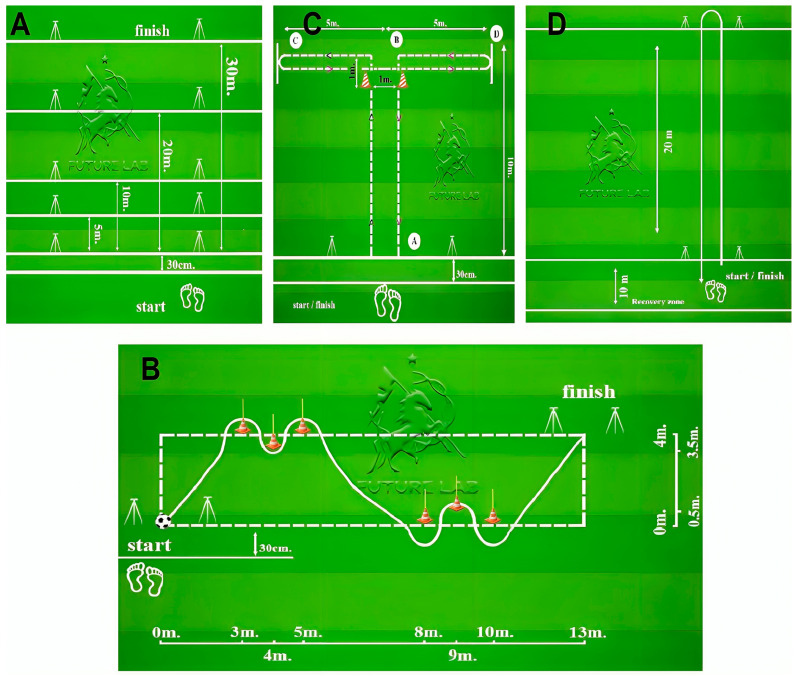
Layout for speed testing—5, 10, 20 and 30 m sprint (**A**), dribbling speed and change of direction and dribbling (**B**), T-drill (**C**) and RSA-test (**D**).

The testing battery included sprinting with splits of 5, 10, 20 and 30 m, the counter-movement jump (CMJ), the change of direction (COD) run, the *T*-test, dribbling and the repeat sprint ability (RSA) test (Figure 1).

After measuring height and body mass before the start of physical and sport-specific tests, all participants, under the supervision of a qualified coach, performed a standardised 12–15 min FIFA 11+ warm-up, which was completed allowing three minutes before the start of the tests to minimise the risk of acute muscle fatigue [17]. All tests, on the control and experimental days, were conducted in a strict sequence.

On the control day, all interventions were conducted in the following sequence:

- Survey using questionnaires (GAD-7, CCQ-r);
- Measurement of height and body mass;
- FIFA 11+ warm-up;
- 30 m sprint with splits of 5-, 10-, 20-m;
- Countermovement jump;
- Change of direction;
- T-drill test;
- Dribbling speed;
- RSA test (6 × 20/20 m with 20 s of rest).

On the experimental day, participants received two capsules (400 mg caffeine or placebo) 60 min before the start of the warm-up exercise. The capsule intake was accompanied by an intake of 150 mL of pure water. Interventions on the experimental day were conducted in the following sequence:

- Measurement of height and body mass
- Caffeine or placebo capsule intake
- FIFA 11+ warm-up;
- 30 m sprint with splits of 5, 10, 20 m;
- Countermovement jump;
- Change of direction;
- T-drill test;
- Dribbling speed;
- RSA test (6 × 20/20 with 20 s of rest).

The rest period between each test was three minutes. Participants were instructed to perform all tests with maximum effort. No verbal encouragement was provided by the study coordinators or coaches during the tests as this has recently been proven to have a positive effect in soccer players [18]. Following the standardised warm-up routine, participants performed two 30 m sprint trials. Between the two efforts, a 3 min recovery period was provided. The best single effort from the straight sprints was used for analysis. Sprint times were recorded using a SmartSpeed Pro timing system (VALD Performance, Brisbane, Australia), with gates at zero, 5, 10, 20 and 30 m. Gates were set at a height of 1 m from the floor. Each attempt was recorded with an accuracy of one hundredth of a second. The SmartSpeed Pro timing system has previously been used to evaluate sprint and agility performances in male and female athletes showing acceptable validity and reliability [19,20].

Participants started all sprint trials from a two-point start position, with their front foot 0.3 m behind the first timing gate, and were instructed to complete with maximum effort. All tests were carried out in specific soccer shoes familiar to the players.

After the jump tests data on CMJ with arms held at the waist were collected. All participants were familiar with the jumping protocols, having completed jumps regularly as part of regular club assessments and participating in several practice testing sessions. All jump tests were conducted at an indoor facility to avoid any external variations in surface that might affect results. In an attempt to standardise jump tests, participants were instructed to perform all attempts in accordance with the protocols outlined by Comrack et al. [21]. For each jump test, three attempts were made and the best result was recorded in centimetres (cm) for further analysis. A recovery interval of 2 min between jumps was provided. A commercially available jump mat (SmartJump™, VALD, Australia) was used to perform the test. The SmartJump system has been previously validated.

Change of direction was assessed using the following test: after stabilising the body position for 2–3 s, the athlete starts moving towards the first pole with the highest possible effort. The athlete runs around the first pole on the left, then the second pole on the right and the third pole on the left. After that, they move to the fourth pole, run around it on the right, the fifth pole on the left and the last pole on the right. The athlete completes the test with maximum acceleration by crossing the finish line. Knocking down the poles or passing hands over them is not allowed; however, the athlete can touch the poles (Figure 1B) [20].

The T-drill test was performed according to the following protocol: the athlete takes the starting position in front of line A, the body is inclined forward, arms are randomly positioned. After stabilising the body position for 2–3 s, the athlete moves forward with the maximum possible effort.

The movement was performed strictly perpendicular between the posts to the poles of point B. Then, the tested athlete turned 90 degrees to the left and started moving towards line C, crossed or stepped on it with one foot. The athlete then turned 180 degrees and ran backwards as fast as possible to the opposite line D, crossed or stepped on it with one foot. They returned to the poles of point B, ran between them and finished in the timing gate at the maximum sprint (Figure 1C) [22].

To assess soccer-specific skills, participants performed the dribbling speed test according to the protocol described by Haugen et al. [20]. The test utilised a SmartSpeed Plus timing system (VALD Performance, Australia), six poles and a standard soccer ball with a pressure of 0.9 atmospheres (Figure 1B). A total of two attempts were performed, with a 3 min recovery period between each attempt. The result of the best attempt was used for further analysis.

When testing for the ability to sprint repeatedly, the participants performed six accelerations of 40 m with maximum effort (two linear distances of 20 m with a 180 degree turn in between) (Figure 1D). After each of the repetitions, the participant would slowly walk back to the starting line and prepare for the next acceleration with a recovery time of 20 s. These test variations closely simulate conditions of a competitive game including changes in movement direction, lactate levels, recovery time and sprint distance [23]. Time of each segment (RSA1–RSA6), total time of all segments (RSAsum), mean time of all segments (RSAmean), time of the best segment (RSAbest), total time of the first and last three segments (RSA1–3 and RSA4–6), the difference between the total time of the first and last three segments (RSA1–3–RSA4–6), fatigue index (FI) and percentage decrement score (Sdec) were evaluated from the test results, with the latter two calculated as described by Girard et al. [24].

### 2.8. Maturity Status

The percentage of predicted adult height determined by the Khamis–Roche formula was used to determine maturity status [25,26]. This method of determining biological maturity is often used in various sporting organisations and in scientific research involving soccer players and can be considered the method of choice for determining the maturity status of young athletes aged 6 to 16 years old [27].

### 2.9. Assessment of Anxiety Levels

The Generalised Anxiety Disorder Screener (GAD-7) Questionnaire, previously used in studies involving soccer players was used to assess the level of anxiety [28,29]. This questionnaire is validated for use in the country [30].

### 2.10. Assessment of Habitual Caffeine Consumption

The Caffeine Consumption Questionnaire-Revised (CCQ-R) previously employed in the Batista et al. study with active lifestyle students was used to assess habitual caffeine consumption [31,32]. The questionnaire was translated and adapted for the study participants.

### 2.11. Assessment of Adverse Events

The incidence of adverse events was assessed using a developed questionnaire that listed the most common adverse events associated with caffeine intake. These included the following symptoms: insomnia, headache, increased urination, gastronintestinal problems, restlessness, irritability, muscle pain, feeling of heart palpitations, tachycardia and/or palpitations. This questionnaire was based on questionnaires used in previous studies on the effects of caffeine on performance [33,34]. Athletes completed the questionnaire 24 h after receiving caffeine capsules or placebo on the experimental day.

### 2.12. Nutritional Aid

The capsules used were white-coloured capsules, each containing either 200 mg of caffeine or placebo (starch), provided by the pharmaceutical company CJSC Evalar. The capsules looked identical and did not differ in colour, shape or taste. The qualitative and quantitative composition of the capsules was verified by high-performance liquid chromatography with ultraviolet detection (HPLC-UV, Agilent 1200, Agilent, Santa Clara, CA, USA) in an independent laboratory.

Before the capsules were administered, they were transferred from the manufacturer’s containers into four identical containers, of which two were numbered and signed as ‘caffeine’ and two as ‘placebo’ according to what was told to each participant group at the time of administration which was necessary to facilitate the deception of the participants. From these containers, the blinded coordinator administrated the capsules according to a randomisation list that included the participants’ identity and the number according to the container. The moment the capsules were administrated to a participant, a coordinator either stated ‘You get caffeine’ or ‘You get placebo’, according to what was written on the container. Each participant was given 2 capsules, totalling 400 mg, which amounted to 4.24–8.64 mg/kg body mass of the participants. No statistical significance was found between groups for this parameter (Table 1).

### 2.13. Ethical Approval

The study was conducted according to the Helsinki declaration and approved by the local ethics committee (N 05-21 dated 10.03.2021). Following a full explanation of the study design and potential risks, all participants and their parents/guardians signed an informed consent to participate in the study.

### 2.14. Statistical Analysis

Statistical analyses were performed using Jamovi 2.2.5 software and Microsoft Excel. The Shapiro–Wilk test was used to assess the normality of variable distribution. Normally (height, mass, BMI, age, CMJ, split 5 and 10 m, COD, RSA total, RSA average, RPE, VAS score after tests) and non-normally (maturity status, slit 20 m and sprint 20 m, dribbling, *t*-test, FI, Sdec, VAS score before tests, GAD, caffeine consumption according to the CCQ-R) distributed data were described using median, minimum and maximum value and interquartile range. Frequency analysis was used to analyse the distribution of players according to age, field position, degree of biological maturation, caffeine consumption and anxiety severity in the four study groups. Fisher’s criterion was used to analyse the incidence of side effects. ANOVA was used to compare pre- and post-caffeine/placebo scores between the four groups for normally distributed variables and Kruskal–Wallace test for non-normal distributions. The one-sample Student’s *t*-test for normally distributed variables and the Wilcoxon test for non-normally distributed variables were used to compare values within each group before and after caffeine/placebo administration. Results were considered statistically significant at *p* < 0.05.

## 3. Results

There were no statistically significant differences in age, body mass, height, BMI and somatic maturation and in habitual caffeine intake (*p* = 0.108) and anxiety level (*p* = 0.875) (Table 2) across all participants. Caffeine consumption according to the CCQ-R questionnaire among all participants was 0–339 mg per day (median: 53.5; IQR: 42.5) and the median anxiety score was 1 (IQR: 2, min–max: 0–8). Only two participants reported conscious experience with caffeine for ergogenic purposes. The examined groups did not statistically differ from each other by the studied indicators on the control day (Table 3).

After testing on the experimental day, statistically significant changes in FI (*p* < 0.001), decrement Sdec (*p* < 0.001) and dribbling speed (*p* = 0.048) were found in group 1. Among group 2 participants, statistically significant changes were observed in 20 m split (*p* = 0.038) and 30 m sprint times (*p* = 0.025). Group 3 participants showed statistically significant changes in RSA5 performance (*p* = 0.029). Participants in group 4 reported statistically significant changes for FI (*p* < 0.001), Sdec (*p* < 0.001), RSA4 and RSA6 (*p* = 0.039 and *p* = 0.005, respectively). There was also a decrease in dribbling speed in group 4 participants (Table 4 and Table 5).

The studied groups did not statistically differ from each other in the analysed parameters on the experimental day, except for FI and Sdec. In the subsequent analysis, it was revealed that in group 4, the FI and Sdec were significantly lower than in group 1 (*p* = 0.039 and *p* = 0.018, respectively).

When analysing the incidence of adverse events during the subsequent 24 h, no statistical significance was found between the groups receiving caffeine or placebo 60 min before physical and sport-specific exercise (Table 6 and Table 7). The groups that received caffeine had a higher incidence of headaches (*p* = 0.019).

## 4. Discussion

Currently, there is sufficiently high evidence to suggest that a single caffeine administration at a dose of 3–6 mg/kg body mass can have a positive effect not only on endurance, but also on sprint and vertical jump performance in team sports [35,36,37]. The results obtained may be related to the previously described direct effect of caffeine on muscles, which is most likely related to the enhancement of contractility due to the mobilisation of calcium ions [38,39,40].

However, data on the caffeine effects on sport-specific skills such as dribbling speed and RSA test of young elite soccer players are still limited. There are only a few studies involving adult soccer players of both sexes with conflicting results. For instance, Del Coso et al., Gant et al. and Foskett et al. demonstrated an increase in total distance covered during match-play, pass accuracy and jump height after caffeine consumption [41,42,43]. Conversely, studies by Astorino et al. and Pettersen et al. that involved female adult and young soccer players did not find improvements in T-test and match activities [44,45]. Notably, only the Pettersen et al. study included young elite soccer players [45].

This study is the first to demonstrate the effect of a single administration of a relatively high dose of caffeine not only on physical performance such as speed and strength, but also on the sport-specific skills of young elite soccer players. An important feature of the study is its design, which allowed us to evaluate the influence of the placebo effect on the obtained results. Another feature is the low habitual consumption of caffeine-containing products by the study participants. Although a number of studies have shown that the habitual high level of caffeine consumption does not affect its ergogenic properties, the influence of this factor cannot be completely excluded [46].

Some of the data obtained in the present study are not supported by previous studies. The first notable finding highlighted the absence of a positive effect of caffeine intake on total RSA test time and best sprint time, which have been previously described in studies involving student-level sprinters, team sport players of low-level athletes [47,48,49]. This may be due to a number of factors including athlete level, testing protocols, training status of study participants and the presence or absence of verbal encouragement, which is known to have an ergogenic effect [50,51]. It should be noted that in previous studies involving soccer players, the participants cannot be classified as elite, which would also affect the results of the tests, as the level of fitness and years of training can undoubtedly influence the magnitude of the effect when testing physical qualities and sport-specific skills that have been trained over a long period of time.

Previously, a number of studies have demonstrated statistically significant placebo effects of caffeine ingestion on parameters important for athletic success such as maximal oxygen consumption, peak speed and peak concentric power [52,53,54]. In these studies, participants were physically active adults who were likely to have a history of positive ergogenic caffeine use. It should be noted that the placebo effects of caffeine may be influenced by factors such as motivation, belief and habitual caffeine consumption [55]. However, the vast majority of participants in this study had not consciously used caffeine or caffeine-containing products for ergogenic purposes previously (only two participants had such experience). Therefore, the absence of a statistically significant effect in the placebo group should not be surprising even though the participants were fully informed prior to the study regarding caffeine and its proven positive effects on athletic performance in various sports.

One of the study highlights is the safety profile of a single-dose of 400 mg caffeine (median: 5.77, IQR: 0.79, min–max: 4.24–8.64 mg/kg) in young athletes. To the authors’ knowledge, this is the first time that such a caffeine administration regimen has been demonstrated to be safe. The findings are consistent with previous studies in adult athletes [11]. It should be noted that long-term caffeine consumption can lead to addiction, insomnia, migraines and other side effects [56]. However, given the advantage it provides in essential soccer performance factors such as RSA, its cautious long-term use may be justified. Limiting consumption to competitive matches may help mitigate these drawbacks while maximising its benefits

### Limitations

The present study is not without limitations that should be considered. The lack of tests that required maximum effort and performed ‘to failure’ (e.g., various modifications of the Yo-Yo tests) was evident as the most pronounced positive effects may have been observed when performing such tests, as the positive effects of caffeine on various aspects of performance have previously been demonstrated under conditions of fatigue [57,58]. Another limitation is the lack of individualisation with regard to the time to reach the maximum serum caffeine concentration, which can vary between 15 and 120 min, as well as the different dose in mg/kg body mass that the participants received [59]. The large variation in participants’ body mass constitutes another limitation of the study, which may have affected the consistency of the results. The administration was based on total body mass rather than lean body mass, which may have influenced the observed outcomes. However, it should be noted that the study protocol allowed 60 min for absorption before exercise, a period commonly used in assessing the effects of caffeine on performance as it corresponds to peak serum concentration [60]. A further limitation is related to the lack of genetic testing for participants to identify single nucleotide polymorphisms (SNPs) that could potentially influence caffeine metabolism and its effects [61,62]. At the same time, there is evidence that the presence of any of these polymorphisms does not affect parameters such as strength, speed and cardiorespiratory parameters following caffeine ingestion [63,64,65].

The main directions for future studies may include the investigation of higher and lower caffeine doses on a range of factors relevant to athletic success among young elite athletes from different sports, as well as the use of caffeine in combination with other dietary supplements frequently utilised by athletes, such as creatine, beta-alanine and sodium bicarbonate [66,67]. In addition, evaluating absorption and maximum plasma concentration would clarify the pharmacokinetics of caffeine in this population and provide a deeper understanding of the results. Another important direction is the investigation of long-term effects of caffeine in elite soccer players to ensure both performance enhancement and athlete well-being. It would also be interesting to study the effect of caffeine in young athletes who already have positive and negative experiences with caffeine intake and with relatively high levels of anxiety.

## 5. Conclusions

A single administration of 400 mg of caffeine 60 min prior to maximal intensity physical activity can be considered reasonable and safe for young soccer players at U15 to U17 with a high degree of somatic maturation. Moreover, there was no placebo effect of caffeine in a youth soccer population with no experience of using this substance for ergogenic purposes.

## Figures and Tables

**Table 1:** Caffeine dosage adjusted for body mass.

	All Participants (n = 54)	Group 1 (n = 14)	Group 2 (n = 12)	Group 3 (n = 15)	Group 4 (n = 13)	*p*-Value
Dose (mg/kg) (Me; IQR; min–max)	5.77; 0.79; 4.24–8.64	5.67; 0.66; 5.12–7.42	5.65; 1.55; 4.24–8.64	5.82; 0.59; 4.82–6.76	5.79; 0.36; 5.38–7.75	0.185

**Table 2:** Anthropometric indicators and somatic maturation of the participants.

	All Participants (n = 54)	Group 1 (n = 14)	Group 2 (n = 12)	Group 3 (n = 15)	**Group 4** **(n = 13)**	***p*-Value**
Age; (Me; IQR; min–max)	16.5; 1.2; 15.1–17.8	16.0; 1.2; 15.1–17.7	16.5; 1.3; 15.2–17.8	16.6; 1.2; 15.1–17.8	16.5; 1.5; 15.5–17.8	0.981
Maturity status (%) (Me; IQR; min–max)	98.6; 2; 91–100	98; 3; 95–100	98; 2.5; 91–99.7	99; 0.9; 95–100	98.6; 2; 95–100	0.818
Height (cm); (Me; IQR; min–max)	180; 9.5; 157–199	182; 7.5; 168–192	182; 20.8; 157–199	179; 6.5; 166–189	180; 6; 170–188	0.846
Body Mass (kg); (Me; IQR; min–max)	69.2; 9.97; 46.4–94.8	70.6; 8.5; 53.9–78.1	71.2; 19.8; 46.4–94.8	68.8; 7; 59.2–82.3	66.7; 4.2; 51.6–73.5	0.554
BMI (kg/m^2^); (Me; IQR; min–max)	21.4; 2.07; 17.9–23.9	21.5; 2.2; 19.1–23.5	22.1; 2.36; 18.8–23.9	21.5; 1.06; 18.8–23.6	21.0; 1.1; 17.9–22.4	0.450

**Table 3:** Results of physical qualities and sport-specific skills tests on the control day. RSA total—total time for all six sprints; FI—fatigue index; Sdec—decrement score of power output.

	Group 1 (n = 14)	Group 2 (n = 12)	Group 3 (n = 15)	**Group 4** **(n = 13)**	***p*-Value**
Split 5 (Me; IQR; min–max)	1.1; 0.04; 0.97–1.16	1.06; 0.09; 1.01–1.16	1.08; 0.04; 0.98–1.14	1.09; 0.1; 0.98–1.26	0.884
Split 10 (Me; IQR; min–max)	1.84; 0.08; 1.7–1.93	1.78; 0.08; 1.71–1.85	1.83; 0.06; 1.74–1.92	1.83; 0.05; 1.72–2.00	0.170
Split 20 (Me; IQR; min–max)	3.08; 0.14; 2.89–3.3	3.03; 0.08; 2.95–3.23	3.04; 0.11; 2.95–3.19	3.05; 0.1; 2.86–3.3	0.517
Sprint 30 (Me; IQR; min–max)	4.25; 0.2; 4.04–4.59	4.17; 0.07; 4.04–4.5	4.22; 0.17; 4.07–4.44	4.19; 0.11; 4.02–4.55	0.693
CMJ (Me; IQR; min–max)	44.7; 5.2; 35.4–56.9	47.1; 7.8; 38.9–53.6	44.4; 3.6; 37.4–56.4	45.2; 6.2; 39.2–60.4	0.968
COD (Me; IQR; min–max)	7.5; 0.4; 7.2–8.4	7.4; 0.4; 7.0–7.9	7.5; 0.3; 7.1–8.0	7.4; 0.4; 6.9–8.1	0.479
T-test (Me; IQR; min–max)	8.5; 0.3; 8.1–9.2	8.3; 0.3; 8.0–8.8	8.4; 0.3; 8.3–8.9	8.3; 0.3; 7.9–9.2	0.064
Dribbling (Me; IQR; min–max)	9.1; 0.4; 8.6–10.2	9.0; 0.6; 8.3–10.3	9.1; 0.3; 8.3–10.5	9.2; 0.5; 8.4–10.1	0.749
RSA total (Me; IQR; min–max)	44.0; 1.7; 42.1–48.2	43.1; 1.6; 41.7–47.9	44.1; 0.9; 42.2–46.9	43.7; 1.9; 41.6–47.1	0.869
RSA mean (Me; IQR; min–max)	7.3; 0.3; 7.0–8.0	7.3; 0.3; 6.9–8.0	7.3; 0.1; 7.0–7.8	7.3; 0.3; 6.9–7.8	0.869
RSA_1_ (Me; IQR; min–max)	7.02; 0.3; 6.69–7.73	6.89; 0.3; 6.73–7.39	7.00; 02; 6.85–7.46	6.83; 0.2; 6.72–7.67	0.113
RSA_2_ (Me; IQR; min–max)	7.17; 0.2; 6.79–8.03	7.13; 0.4; 5.29–7.55	7.22; 0.2; 6.84–7.71	7.11; 0.4; 6.83–7.72	0.645
RSA_3_ (Me; IQR; min–max)	7.36; 0.4; 6.94–8.12	7.34; 0.4; 6.88–7.96	7.39; 0.2; 6.89–7.85	7.22; 0.5; 6.76–7.66	0.746
RSA_4_ (Me; IQR; min–max)	7.56; 0.3; 6.94–8.01	7.36; 0.3; 7.01–8.11	7.45; 0.3; 6.98–7.85	7.51; 0.4; 7.00–8.04	0.921
RSA_5_ (Me; IQR; min–max)	7.59; 0.3; 7.09–8.20	7.32; 0.4; 7.04–8.68	7.56; 0.2; 7.25–7.96	7.54; 0.3; 7.06–8.17	0.328
RSA_6_ (Me; IQR; min–max)	7.51; 0.5; 7.21–8.09	7.52; 0.4; 7.11–8.87	7.53; 0.2; 7.23–8.12	7.62; 0.4; 7.20–8.28	0.476
RSA best (Me; IQR; min–max)	7.02; 0.279; 6.69–7.73	6.88; 0.4; 5.29–7.39	6.95; 0.2; 6.84–7.46	6.83; 0.2; 6.72–7.66	0.137
RSA_1–3_ (Me; IQR; min–max)	21.6; 1; 20.6–23.9	21.2; 1; 19.4–22.9	21.6; 0.6; 20.6–23.0	21.2; 1; 20.3; 23.1	0.591
RSA_4–6_ (Me; IQR; min–max)	22.8; 0.7; 21.2–24.3	22.1; 0.764; 21.2–25.5	22.5; 0.6; 21.6–23.9	22.5; 0.6; 21.6–23.9	0.350
RSA_1–3_–RSA_4–6_ (Me; IQR; min–max)	−0.93; 0.7; −2.05–−0.352	−0.82; 0.7; −3.16–0.121	−0.99; 0.4; −1.53–0.028	−0.938; 0.3; −1.53–0.028	0.050
FI (Me; IQR; min–max)	7.9; 3.4; 4.0–15.6	9.2; 5.2; 4.2–45.1	8.6; 2.8; 4.8–11.8	10.2; 1.8; 5.0–20.1	0.257
Sdec (Me; IQR; min–max)	4.5; 1.3; 1.2–7.5	4.6; 2.6; 2.1–31.9	4.7; 2.3; 2.6–7.2	6.1; 1.7; 2.5–9.4	0.251

**Table 4:** Results of speed, strength, dribbling and change of direction tests on control and experimental days among participants. CMJ—countermovement jump, COD—change of direction test.

	Group 1 (n = 14)		Group 2 (n = 12)		Group 3 (n = 15)		**Group 4** **(n = 13)**	
	Contr. day	Exp. day	Contr. day	Exp. day	Contr. day	Exp. day	Contr. day	Exp. day
Split 5 (Me; IQR; min–max)	1.1; 0.04; 0.97–1.16	1.04; 0.12; 0.97–1.93	1.06; 0.09; 1.01–1.16	1.08; 0.08; 1.0–1.2	1.08; 0.04; 0.98–1.14	1.09; 0.1; 0.99–1.2	1.09; 0.1; 0.98–1.26	1.02; 0.05; 0.98–1.21
*p*-value	0.086		0.553		0.155		0.080	
Split 10 (Me; IQR; min–max)	1.84; 0.08; 1.7–1.93	1.79; 0.11; 1.67–1.97	1.78; 0.08; 1.71–1.85	1.83; 0.11; 1.73–1.92	1.83; 0.06; 1.74–1.92	1.83; 0.11; 1.75–1.94	1.83; 0.05; 1.72–2.00	1.78; 0.09; 1.7–1.91
*p*-value	0.137		0.075		0.405		0.054	
Split 20 (Me; IQR; min–max)	3.08; 0.14; 2.89–3.3	3.05; 0.17; 2.87–3.28	3.03; 0.08; 2.95–3.23	3.06; 0.09; 2.96–3.25	3.04; 0.11; 2.95–3.19	3.04; 0.17; 2.96–3.25	3.05; 0.1; 2.86–3.3	3.04; 0.15; 2.87–3.26
*p*-value	0.073		0.038		0.201		0.198	
Sprint 30 (Me; IQR; min–max)	4.25; 0.2; 4.04–4.59	4.22; 0.24; 3.96–4.57	4.17; 0.07; 4.04–4.5	4.23; 0.09; 4.1–4.61	4.22; 0.17; 4.07–4.44	4.2; 0.22; 4.05–4.53	4.19; 0.11; 4.02–4.55	4.18; 0.2; 3.96–4.6
*p*-value	0.077		0.025		0.229		0.152	
CMJ (Me; IQR; min–max)	44.7; 5.2; 35.4–56.9	45.5; 6.3; 35.1–56.4	47.1; 7.8; 38.9–53.6	45.7; 5.2; 38.7–56.0	44.4; 3.6; 37.4–56.4	44.4; 6.3; 38.6–54.7	45.2; 6.2; 39.2–60.4	44.4; 5.4; 38.2–61.5
*p*-value	0.929		0.818		0.631		0.631	
COD (Me; IQR; min–max)	7.5; 0.4; 7.2–8.4	7.4; 0.4; 7.1–7.9	7.4; 0.4; 7.0–7.9	7.4; 0.4; 7.0–7.9	7.5; 0.3; 7.1–8.0	7.5; 0.2; 7.1–8.0	7.4; 0.4; 6.9–8.1	7.4; 0.4; 6.9–8.1
*p*-value	0.126		0.479		0.683		0.949	
*T*-test (Me; IQR; min–max)	8.5; 0.3; 8.1–9.2	8.5; 0.3; 8.0–9.0	8.3; 0.3; 8.0–8.8	8.4; 0.4; 7.9–8.7	8.4; 0.3; 8.3–8.9	8.4; 0.3; 8.0–8.7	8.3; 0.3; 7.9–9.2	8.3; 0.2; 7.8–9.0
*p*-value	0.175		0.519		0.476		0.542	
Dribbling (Me; IQR; min–max)	9.1; 0.4; 8.6–10.2	8.9; 0.4; 8.1–10.0	9.0; 0.6; 8.3–10.3	9.0; 0.6; 7.9–9.9	9.1; 0.3; 8.3–10.5	9.1; 0.4; 8.4–10.1	9.2; 0.5; 8.4–10.1	8.8; 0.5; 8.4–10.0
*p*-value	*0.048*		0.228		0.679		0.064	

**Table 5:** Results of repeated sprint ability test on control and experimental days among participants. RSA total—total time for all six sprints; FI—fatigue index; Sdec—decrement score of power output.

	Group 1 (n = 14)		Group 2 (n = 12)		Group 3 (n = 15)		**Group 4** **(n = 13)**	
	Contr. day	Exp. day	Contr. day	Exp. day	Contr. day	Exp. day	Contr. day	Exp. day
RSA total (Me; IQR; min–max)	44.0; 1.7; 42.1–48.2	44.2; 1.4; 41.9–46.6	43.1; 1.6; 41.7–47.9	44.1; 2.1; 42.5–47.0	44.1; 0.9; 42.2–46.9	43.9; 1.0; 42.1–46.7	43.7; 1.9; 41.6–47.1	44.0; 1.5; 40.8–46.3
*p*	0.226		0.054		0.178		0.226	
RSA mean (Me; IQR; min–max)	7.3; 0.3; 7.0–8.0	7.4; 0.2; 7.0–7.8	7.3; 0.3; 6.9–8.0	7.2; 0.3; 7.1–7.8	7.3; 0.1; 7.0–7.8	7.3; 0.2; 7.0–7.8	7.3; 0.3; 6.9–7.8	7.3; 0.2; 6.8–7.7
*p*-value	0.226		0.055		0.178		0.226	
RSA_1_ (Me; IQR; min–max)	7.02; 0.3; 6.69–7.73	7.03; 0.3; 6.70–7.44	6.89; 0.3; 6.73–7.39	7.09; 0.3; 6.68–7.31	7.00; 02; 6.85–7.46	7.03; 0.3; 6.70–7.57	6.83; 0.2; 6.72–7.67	7.03; 0.3; 6.70–7.57
*p*-value	0.538		0.163		0.258		0.057	
RSA_2_ (Me; IQR; min–max)	7.17; 0.2; 6.79–8.03	7.19; 0.3; 6.88–7.64	7.13; 0.4; 5.29–7.55	7.17; 0.3; 6.96–7.53	7.22; 0.2; 6.84–7.71	7.2; 0.2; 6.85–7.67	7.11; 0.4; 6.83–7.72	7.20; 0.2; 6.85–7.67
*p*-value	0.321		0.092		0.369		0.685	
RSA_3_ (Me; IQR; min–max)	7.36; 0.4; 6.94–8.12	7.36; 0.4; 6.95–7.72	7.34; 0.4; 6.88–7.96	7.39; 0.3; 6.99–7.59	7.39; 0.2; 6.89–7.85	7.32; 0.2; 6.83–7.89	7.22; 0.5; 6.76–7.66	7.32; 0.2; 6.83–7.89
*p*-value	0.584		0.783		0.684		0.962	
RSA_4_ (Me; IQR; min–max)	7.56; 0.3; 6.94–8.01	7.45; 0.5; 6.98–7.87	7.36; 0.3; 7.01–8.11	7.42; 0.4; 7.19–8.19	7.45; 0.3; 6.98–7.85	7.43; 0.2; 6.80–7.91	7.51; 0.4; 7.00–8.04	7.43; 0.2; 6.80–7.91
*p*-value	0.079		0.266		0.523		0.039	
RSA_5_ (Me; IQR; min–max)	7.59; 0.3; 7.09–8.20	7.46; 0.3; 7.07–7.96	7.32; 0.4; 7.04–8.68	7.51; 0.4; 7.28–8.46	7.56; 0.2; 7.25–7.96	7.43; 0.3; 7.18–7.78	7.54; 0.3; 7.06–8.17	7.43; 0.4; 7.18–7.78
*p*-value	0.069		0.065		0.029		0.156	
RSA_6_ (Me; IQR; min–max)	7.51; 0.5; 7.21–8.09	7.44; 0.5; 7.06–8.07	7.52; 0.4; 7.11–8.87	7.55; 0.4; 7.24–8.33	7.53; 0.2; 7.23–8.12	7.55; 0.1; 7.10–7.88	7.62; 0.4; 7.20–8.28	7.55; 0.1; 7.10–7.88
*p*-value	0.220		0.519		0.992		0.005	
RSA best (Me; IQR; min–max)	7.02; 0.279; 6.69–7.73	7.03; 0.3; 6.70–7.38	6.88; 0.4; 5.29–7.39	7.09; 0.3; 6.68–7.31	6.95; 0.2; 6.84–7.46	7.0; 0.3; 6.70–7.57	6.83; 0.2; 6.72–7.66	7.07; 0.3; 6.60–7.55
*p*-value	0.476		0.064		0.489		0.057	
RSA_1–3_ (Me; IQR; min–max)	21.6; 1; 20.6–23.9	21.6; 0.9; 20.6–22.7	21.2; 1; 19.4–22.9	21.7; 0.9; 20.6–22.4	21.6; 0.6; 20.6–23.0	21.7; 0.7; 20.4–23.1	21.2; 1; 20.3; 23.1	21.7; 0.6; 20.4–23.1
*p*-value	0.428		0.146		0.313		0.328	
RSA_4–6_ (Me; IQR; min–max)	22.8; 0.7; 21.2–24.3	22.4; 0.9; 21.3–23.9	22.1; 0.764; 21.2–25.5	22.4; 1.1; 21.9–25.0	22.5; 0.6; 21.6–23.9	22.4; 0.3; 21.6–23.6	22.5; 0.6; 21.6–23.9	22.4; 0.2; 21.6–23.6
*p*-value	0.05		0.110		0.273		0.479	
RSA_1–3_–RSA_4–6_ (Me; IQR; min–max)	−0.93; 0.7; −2.05–−0.352	−0.85; 0.6; −1.52–−0.237	−0.82; 0.7; −3.16–0.121	−0.94; 0.5; −2.95–−0.64	−0.99; 0.4; −1.53–0.028	−0.829; 0.5; −1.93–−0.05	−0.938; 0.3; −1.53–0.028	−0.79; 0.6; −1.93–−0.05
*p*-value	0.385		0.733		0.742		0.860	
FI (Me; IQR; min–max)	7.9; 3.4; 4.0–15.6	7.4; 2.2; 2.2–12.9	9.2; 5.2; 4.2–45.1	8.0; 2.3; 5.6–17.2	8.6; 2.8; 4.8–11.8	8.0; 4.1; 4.5–16.7	10.2; 1.8; 5.0–20.1	6.7; 3.8; 3.5–12.0
*p*-value	<0.001		0.266		0.847		< 0.001	
Sdec (Me; IQR; min–max)	4.5; 1.3; 1.2–7.5	4.2; 1.3; 1.3–6.8	4.6; 2.6; 2.1–31.9	4.7; 1.7; 2.2–8.5	4.7; 2.3; 2.6–7.2	4.9; 1.4; 2.8–7.6	6.1; 1.7; 2.5–9.4	3.3; 1.2; 1.5–7.3
*p*-value	<0.001		0.301		0.867		<0.001	

**Table 6:** The incidence of adverse events among participants in all four groups within 24 h after administration of 400 mg caffeine or placebo.

	Group 1 (n = 14)	Group 2 (n = 12)	Group 3 (n = 15)	Group 4 (n = 13)	*p*-Value
Insomnia	1 (7.1%)	0 (0%)	1 (6.6%)	1 (7.7%)	0.82
Increased urine production	0	0	0	1 (7.7%)	-
Gastrointestinal problems	2 (14.3%)	2 (16.7%)	0	1 (7.7%)	0.82
Restlessness	4 (28.6%)	3 (25%)	1 (6.6%)	3 (23.1%)	0.47
Headache	3 (21.4%)	0	0	2 (15.4%)	0.67
Irritability	0	0	1 (6.6%)	3 (23.1%)	0.22
Muscular pain	3 (21.4%)	3 (25%)	5 (33.3%)	2 (15.4%)	0.73
Tachycardia/palpitations	0	0	0	1 (7.7%)	-
Number of participants with at least one adverse event	9	5	6	6	0.56

**Table 7:** Comparison of the adverse event incidence within 24 h between the groups receiving caffeine at a dose of 400 mg or placebo.

	Group 1 and 4	Group 2 and 3	*p*-Value
Insomnia	2 (7.4%)	0	0.15
Increased urine production	1 (3.7%)	0	0.30
Gastrointestinal problems	3 (11.1%)	2 (7.4%)	0.64
Increased activeness	7 (25.9%)	4 (14.8%)	0.31
Headache	5 (18.5%)	0	0.019
Irritability	3 (11.1%)	1 (3.7%)	0.30
Muscular pain	5 (18.5%)	8 (29.6%)	0.34
Tachycardia/palpitations	1 (3.7%)	0	0.30
Number of participants with at least one adverse event	15 (55.5%)	11 (40.7%)	0.28

## Data Availability

The datasets used and/or analysed during the current study are available from the corresponding author on reasonable request.
